# Orbital lymphangioma mimicking cellulitis: Diagnostic contribution of MRI and successful management with bleomycin sclerotherapy

**DOI:** 10.1016/j.radcr.2026.05.007

**Published:** 2026-06-04

**Authors:** Mohamed Bouallou, Issam Berrajaa, M’barek Ben Youssef, Achraf Amine Sbai, Drissia Benfadil, Azeddine Lachkar

**Affiliations:** a Department of Otorhinolaryngology, Mohammed VI University Hospital, Oujda, 60000, Morocco; b Faculty of Medicine and Pharmacy, Mohammed First University, Oujda, 60000, Morocco; c Mohammed First University, Oujda, 60000, Morocco

**Keywords:** Orbital lymphangioma, Orbital cellulitis, Magnetic resonance imaging, Sclerotherapy, Bleomycin

## Abstract

Orbital lymphangioma is a rare benign low-flow venous–lymphatic malformation that typically presents with slowly progressive proptosis in children. Acute inflammatory presentations mimicking orbital cellulitis are uncommon and may lead to diagnostic delay. We report the case of an 11-year-old girl presenting with acute left-sided facial swelling and proptosis following an upper respiratory tract infection. Initial management for presumed orbital cellulitis with intravenous antibiotic therapy was unsuccessful. Contrast-enhanced CT showed diffuse soft tissue infiltration without abscess formation, while MRI revealed a multiloculated cystic lesion with hemorrhagic components involving both intra- and extraconal spaces, consistent with orbital lymphangioma. The patient underwent 3 sessions of bleomycin-based sclerotherapy at 1-month intervals, with good tolerance and no complications. Progressive clinical improvement was observed, with complete resolution of proptosis and chemosis at 5 months and no recurrence at 1-year follow-up. This case highlights the key role of MRI in differentiating orbital lymphangioma from infectious processes and supports sclerotherapy as an effective minimally invasive treatment option.

## Introduction

Orbital lymphangioma, currently classified as a low-flow venous–lymphatic malformation according to the International Society for the Study of Vascular Anomalies (ISSVA) 2018 classification, is a rare benign orbital lesion characterized by infiltrative growth and absence of a true capsule [1]. Despite its benign nature, it may lead to significant functional impairment due to its tendency to involve critical orbital structures and to undergo episodic intralesional hemorrhage.

These lesions predominantly affect children and typically present with slowly progressive proptosis. However, acute presentations may occur, most often related to hemorrhagic events or, less commonly, associated inflammatory changes [2]. In such cases, orbital lymphangioma may closely mimic orbital cellulitis, creating a diagnostic dilemma and potentially delaying appropriate management.

Imaging, particularly magnetic resonance imaging (MRI), plays a crucial role in distinguishing lymphangiomas from other orbital pathologies by demonstrating their characteristic multiloculated architecture and hemorrhagic components [3].

We report a pediatric case of orbital lymphangioma presenting with acute inflammatory features suggestive of orbital cellulitis. This case underscores the importance of considering vascular malformations in atypical or treatment-resistant orbital infections and highlights the role of MRI in guiding diagnosis and management, as well as the effectiveness of bleomycin-based sclerotherapy as a minimally invasive therapeutic option.

## Case presentation

An 11-year-old girl was referred to our Department of Otorhinolaryngology for evaluation of a progressive left-sided facial swelling associated with orbital involvement evolving over the previous 7 days (Fig. 1). The swelling initially appeared in the left jugal region and progressively increased in size, accompanied by left-sided orbital proptosis. There was a recent history of an upper respiratory tract infection. There was no history of insect bite, facial trauma, or recent dental extraction, nor any underlying systemic disease such as diabetes mellitus.

**Fig. 1:**
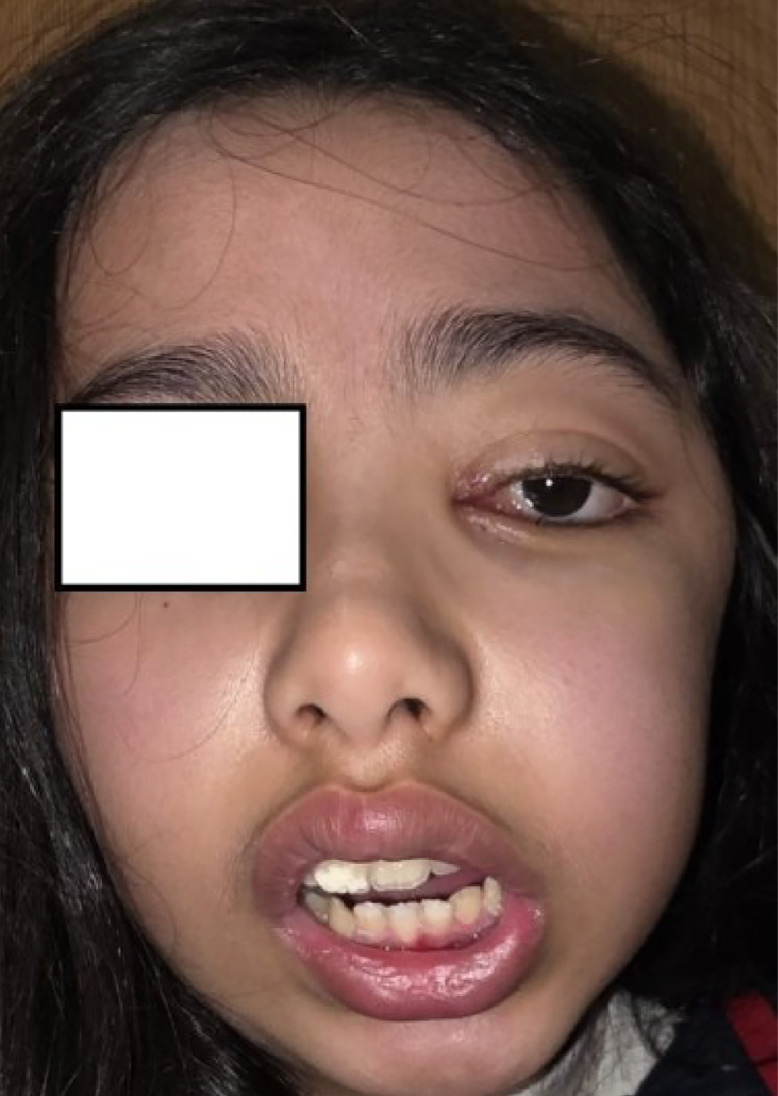
Clinical appearance demonstrating left-sided proptosis associated with an extensive erythematous left jugal swelling extending toward the orbit.

The patient had initially been evaluated by a general practitioner, where a diagnosis of orbital cellulitis was suspected. She received empirical treatment with intravenous amoxicillin–clavulanic acid at a dose of 80 mg/kg/day for 5 days. However, due to persistence of symptoms without significant clinical improvement, the patient was referred to our tertiary care center for further evaluation.

On admission, the patient was conscious, afebrile, and hemodynamically stable. Otorhinolaryngological examination revealed a left jugal swelling with overlying erythema, warmth, and tenderness on palpation. The lesion had a soft consistency and poorly defined margins. Examination of the oral cavity was unremarkable. Nasal endoscopy demonstrated inflammatory changes of the nasal mucosa associated with inferior turbinate hypertrophy, while the middle and inferior meatuses remained patent. Cervical examination did not reveal palpable lymphadenopathy.

Ophthalmologic examination showed unilateral left-sided proptosis associated with conjunctival hyperemia and chemosis. Ocular motility was restricted in the affected eye. Best-corrected visual acuity was reduced to 7/10 on the left side. Fundoscopic examination revealed vascular tortuosity of the retinal vessels without signs of papilledema. Examination of the contralateral eye was normal. The remainder of the systemic examination was unremarkable.

Laboratory investigations revealed mild leukocytosis with a white blood cell count of 11 × 10³/mm³ (reference range: 3.0-10.0 × 10³/mm³), a hemoglobin level of 15.7 g/dL, and a platelet count of 279 × 10³/mm³. The C-reactive protein level was elevated at 41 mg/L (Normal: 7 mg/L).

An emergency contrast-enhanced CT scan of the head and neck demonstrated marked infiltration of the left jugal soft tissues extending toward the orbital apex and intraconal fat, associated with reactive laterocervical lymphadenopathy, the largest measuring 22 mm in short-axis diameter, with no evidence of abscess formation, suggesting either orbital cellulitis or an inflammatory pseudotumor (Fig. 2).

**Fig. 2:**
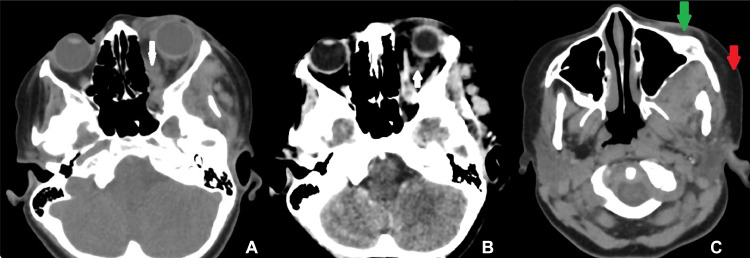
Head and neck CT findings: (A–B) Axial CT images in the soft-tissue window demonstrating infiltration of the intraconal orbital fat (white arrow) (A), without significant enhancement after contrast administration (B). (C) Axial CT image in the soft-tissue window showing associated infiltration of the left jugal subcutaneous tissues (green arrow) and the left temporal soft tissues (red arrow), without evidence of a drainable abscess collection.

For further characterization of the orbital lesion, orbital MRI was subsequently performed. MRI revealed a multiloculated lesion consistent with a vascular malformation with predominant lymphatic components. The lesion demonstrated hyperintense signal on T1-weighted sequences, suggestive of intralesional hemorrhagic changes, and marked hyperintensity on T2-weighted sequences. Multiple thin internal septations were identified, showing enhancement after contrast administration. The lesion measured approximately 32 × 56 mm with a craniocaudal extension of about 30 mm. It involved both the intraconal and extraconal compartments, with encasement of the optic nerve and extension toward the intracavernous segment of the internal carotid artery, resulting in grade III proptosis (Fig. 3).

**Fig. 3:**
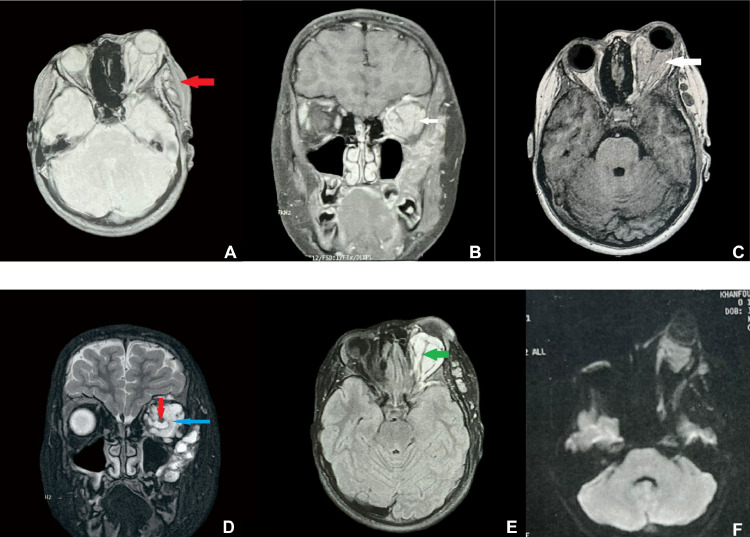
Orbital MRI findings in the present case: (A) Axial non–contrast-enhanced T1-weighted image demonstrating a left orbital cystic lesion with predominantly isointense signal intensity and a multiloculated appearance, associated with thickening of the left temporoparietal soft tissues (red arrow). (B–C) Post-contrast T1-weighted images in coronal (B) and axial (C) planes showing a poorly defined multiloculated cystic mass in the left orbit (white arrow). The lesion contains multiple internal septations demonstrating marked enhancement after gadolinium administration, with peripheral enhancement of the cystic components. **(D)** Coronal T2-weighted fast spin-echo (FSE) sequence demonstrating a markedly hyperintense multiloculated cystic lesion in the left orbit (blue arrow), containing internal areas of low signal intensity compatible with hemorrhagic components (red arrow). **(E)** Axial T2-FLAIR image demonstrating a hyperintense cystic lesion exerting mass effect on the left optic nerve (green arrow) along its intraorbital and intracanalicular segments. **(F)** Axial diffusion-weighted imaging demonstrating a heterogeneous hyperintense multicystic lesion within the left orbit involving both the intraconal and extraconal compartments.

Following multidisciplinary discussion, the diagnosis of orbital lymphangioma was established. The patient continued treatment with intravenous amoxicillin–clavulanic acid (80 mg/kg/day) for 7 days until normalization of inflammatory markers.

Definitive management of the lymphatic malformation consisted of bleomycin-based sclerotherapy. A total of 3 treatment sessions were administered at 1-month intervals. The procedure was well tolerated, with no reported complications or adverse effects.

The patient underwent regular monthly follow-up with comprehensive otorhinolaryngological and ophthalmological examinations. Following the third sclerotherapy session, a significant reduction in proptosis and conjunctival chemosis was observed. Follow-up evaluation included ophthalmologic examination, fundoscopic assessment, complete ENT examination, and orbital MRI.

Progressive clinical improvement was observed during follow-up. Complete resolution of pain, proptosis, and conjunctival chemosis was achieved 5 months after the final sclerotherapy session. At 1-year follow-up, the patient remained asymptomatic, with no evidence of recurrence (Fig. 4).

**Fig. 4:**
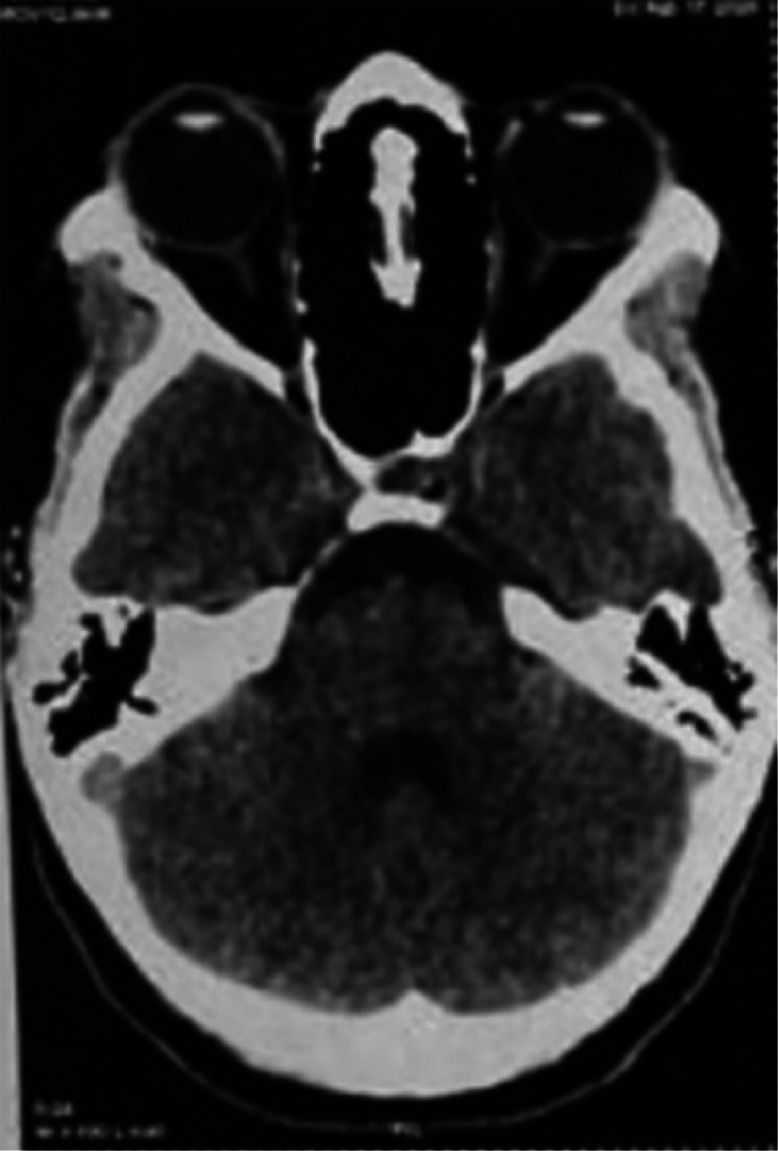
Axial head and neck CT image in the soft-tissue window obtained one year after sclerotherapy, demonstrating complete regression of the orbital cystic mass.

## Discussion

Orbital lymphangioma, currently classified within the spectrum of low-flow venous–lymphatic malformations according to the ISSVA classification, represents a rare subset of orbital vascular anomalies, accounting for approximately 1%-4% of orbital tumors [4]. These lesions are congenital, benign, and non-encapsulated vascular malformations composed of dilated lymphatic channels embedded within a fibrovascular stroma.

Lymphangiomas are relatively uncommon congenital vascular malformations, with an estimated incidence of 1.1-5.3 cases per 10,000 live births. In the pediatric population, they represent a considerable proportion of benign vascular tumors, accounting for approximately 25% of cases and about 4% of all vascular tumors overall [5].

Orbital lymphangiomas predominantly affect the pediatric population, with most cases diagnosed during the first 2 decades of life [6]. Congenital lymphangiomas generally present during early childhood, most often before the age of 5, and are thought to originate from abnormal embryologic development of the lymphatic system leading to incomplete communication with the primary lymphatic channels. Conversely, acquired forms may develop later in life as a consequence of conditions that impair lymphatic drainage, such as malignancy, radiation therapy, trauma, or prior surgical procedures [7].

Clinically, orbital lymphangiomas most commonly present with progressive proptosis, resulting from the slow and insidious expansion of the malformation within the confined orbital cavity [8]. However, the clinical course is often unpredictable and may be punctuated by episodes of intralesional hemorrhage, which may lead to a sudden increase in lesion volume and acute worsening of orbital symptoms. Such hemorrhagic events can cause rapid orbital expansion, orbital pain, chemosis, and varying degrees of visual impairment.

The resulting mass effect may exert significant pressure on adjacent orbital structures, particularly the optic nerve, potentially leading to compressive optic neuropathy. In pediatric patients, prolonged optic nerve compression or distortion of the visual axis may result in amblyopia, thereby compromising long-term visual development. In addition, secondary inflammatory changes may occasionally obscure the underlying vascular malformation and mimic other orbital pathologies.

Acute presentations of orbital lymphangioma mimicking orbital cellulitis are uncommon and represent a diagnostic challenge. Although these lesions typically follow a chronic and indolent course, inflammatory or hemorrhagic events may lead to sudden symptom onset, closely resembling an infectious process. Only a limited number of such pseudo-cellulitic presentations have been reported in the literature, including a case described by Riaz et al. [9] involving a 5-year-old child initially diagnosed with orbital cellulitis.

Several pediatric reports have highlighted the diagnostic contribution of MRI and the effectiveness of bleomycin sclerotherapy in the management of orbital lymphangiomas. A chronological synthesis of the main clinical, radiological, and therapeutic features reported in the literature, culminating with the present case, is provided in Table 1. Despite these reports, acute inflammatory presentations remain uncommon and may lead to diagnostic delay, particularly in cases with poor response to empirical antibiotic therapy.

**Table 1:** Chronological overview of reported pediatric cases of orbital lymphangioma with MRI characterization and bleomycin-based sclerotherapy.

Study	Year	Patient age	Clinical presentation	MRI findings	Treatment	Outcome
Lee et al. [10]	2015	Pediatric	Orbital swelling / proptosis	Multiloculated cystic lesion with hemorrhagic components	Bleomycin + drainage	Favorable outcome
Aristiady et al. [11]	2023	Pediatric	Progressive proptosis	Multiloculated cystic lesion with internal septations	Bleomycin sclerotherapy	Significant regression
Tanveer et al. [12]	2025	Pediatric	Proptosis	Multicystic orbital lesion	Bleomycin sclerotherapy	Clinical improvement
Present case	2026	Pediatric	Acute pseudo-cellulitic presentation with proptosis	Multiloculated cystic lesion with hemorrhagic components involving intra- and extraconal spaces	Bleomycin sclerotherapy (3 sessions)	Complete resolution at 5 months, no recurrence at 1 year

In this context, our case further expands the existing literature by illustrating a pseudo-cellulitic presentation associated with intralesional hemorrhage occurring in the setting of a recent upper respiratory tract infection, thereby underscoring the potential for diagnostic confusion and the critical role of imaging in establishing the correct diagnosis.

Imaging plays a pivotal role in the evaluation of orbital lymphangiomas and is essential for both diagnostic characterization and therapeutic planning. CT may demonstrate a poorly circumscribed cystic or heterogeneous orbital mass with mild enhancement and occasional phleboliths reflecting a venous component [13]. However, magnetic resonance imaging remains the modality of choice owing to its superior soft-tissue contrast resolution and multiplanar capability.

On MRI, orbital lymphangiomas typically appear as multiloculated cystic lesions involving the intraconal and/or extraconal compartments, frequently crossing normal fascial planes, which reflects the infiltrative nature of these malformations. The MRI signal characteristics of orbital lymphangiomas are heterogeneous and largely depend on the presence and the chronological stage of intralesional hemorrhage. In most cases, the lesion demonstrates signal intensity that is iso- to mildly hyperintense relative to brain parenchyma on T1-weighted images, while appearing markedly hyperintense on T2-weighted sequences, reflecting its predominantly fluid-filled cystic architecture.

Internal septations are frequently observed, contributing to the typical multiloculated appearance of the lesion. Variations in both T1 and T2 signal intensities may occur according to the age and composition of blood degradation products within the cystic spaces, sometimes producing characteristic fluid–fluid levels [3]. After contrast administration, the venous component of the malformation typically shows enhancement, whereas the lymphatic component usually demonstrates only subtle enhancement of the thin internal septa, consistent with the low-flow nature of this vascular malformation.

In the radiologic evaluation of orbital masses, orbital lymphangioma may occasionally be misinterpreted as other vascular or neoplastic lesions, particularly when imaging findings are heterogeneous or when hemorrhagic complications obscure the typical multiloculated architecture. The main differential diagnoses include orbital hemangioma, rhabdomyosarcoma, and orbital lymphoma, each of which presents with distinct clinical and imaging characteristics that should be carefully considered.

Orbital hemangioma, more precisely referred to as a cavernous venous malformation, represents the most common benign orbital tumor in adults. Unlike orbital lymphangioma, cavernous hemangiomas are typically well-circumscribed, encapsulated lesions located within the intraconal space. On imaging, they usually appear as homogeneous masses with smooth margins and demonstrate progressive and uniform contrast enhancement following contrast administration [14]. In contrast, orbital lymphangiomas are infiltrative, non-encapsulated lesions that frequently extend across orbital compartments and demonstrate a multiloculated cystic architecture with internal septations and fluid–fluid levels related to previous hemorrhagic events. In our case, the lesion demonstrated a heterogeneous multicystic appearance with hemorrhagic components rather than a well-defined enhancing mass, making a cavernous hemangioma less likely.

Another important diagnostic consideration is rhabdomyosarcoma, the most common primary malignant orbital tumor in children. Clinically, rhabdomyosarcoma typically presents with rapidly progressive unilateral proptosis, frequently accompanied by pain, eyelid swelling, and sometimes diplopia [15]. Radiologically, this tumor usually appears as a solid, aggressive soft-tissue mass, often demonstrating marked contrast enhancement and potential bone remodeling or destruction [16]. Unlike lymphangiomas, rhabdomyosarcomas are generally solid rather than cystic and lack the characteristic multiloculated architecture with hemorrhagic fluid levels. Furthermore, rhabdomyosarcoma tends to exhibit rapid clinical progression, whereas lymphangiomas often follow a more indolent course punctuated by episodic hemorrhage. In our observation, the presence of a multiloculated cystic lesion with variable signal intensities on MRI, reflecting hemorrhagic products of different ages, strongly favored a lymphatic malformation over a malignant neoplasm.

Orbital dermoid cysts also constitute an important differential diagnosis, as they represent one of the most common benign congenital orbital lesions in children and young adults. Dermoid cysts typically arise along craniofacial suture lines, most frequently at the frontozygomatic region, and appear on imaging as well-circumscribed, encapsulated lesions that often contain fat density or signal, a feature highly suggestive of this diagnosis [17].

The management of orbital lymphangiomas remains challenging because of their infiltrative growth pattern and the absence of a well-defined capsule, which often precludes complete surgical excision. Therapeutic strategies must therefore be individualized and depend on lesion size, anatomical location, and the extent of involvement of adjacent orbital structures [9].

When complete surgical excision is not feasible, particularly due to the proximity of the lesion to optic nerve, intralesional sclerotherapy has emerged as an effective minimally invasive therapeutic option [11]. Compared with surgical management, sclerotherapy offers several advantages, including technical simplicity, the possibility of repeated treatments, and a reduced risk of injury to adjacent neurovascular structures [12].

Several sclerosants have been used in the treatment of lymphatic malformations, including bleomycin, ethanol, OK-432, doxycycline, hypertonic saline, acetic acid, and sodium tetradecyl sulfate. Among these agents, bleomycin is widely used because of its potent intralesional sclerosing effect on abnormal vascular endothelium as well as its antiangiogenic properties [18].

In the present case, the patient underwent 3 sessions of intralesional bleomycin sclerotherapy, resulting in progressive reduction of the lesion and complete resolution of proptosis and chemosis within approximately 5 months. This favorable outcome underscores the efficacy of sclerotherapy in managing deep-seated and hemorrhagic orbital lymphangiomas, which are often challenging to treat surgically due to their diffuse infiltration and proximity to critical orbital structures. The results observed in our patient are consistent with multiple reports in the literature, which have demonstrated that bleomycin sclerotherapy can achieve significant clinical improvement, even in refractory or extensive lesions, with a low risk of complications [18,19].

This report is limited by its single-case design, which restricts the generalizability of the findings. The follow-up period remains relatively short and may not fully capture the risk of late recurrence. In addition, the absence of histopathological confirmation may be considered a limitation, although the diagnosis was strongly supported by imaging features and clinical evolution.

The present case is notable for its atypical presentation mimicking orbital cellulitis, which initially suggested an infectious orbital process due to the presence of acute proptosis, eyelid edema, and inflammatory signs. However, imaging revealed a multicystic lesion with hemorrhagic components, consistent with orbital lymphangioma. Such atypical presentations remain uncommon and have been occasionally reported in the literature. This case highlights the importance of considering vascular malformations in pseudo-cellulitic orbital presentations and underscores the key role of MRI in establishing the correct diagnosis and guiding management.

## Conclusion

Orbital lymphangioma is a rare vascular malformation that may exceptionally present with acute inflammatory features mimicking orbital cellulitis, leading to potential diagnostic delay. This case highlights the pivotal role of MRI in establishing an accurate diagnosis in atypical or treatment-resistant presentations. Bleomycin-based sclerotherapy represents an effective and safe minimally invasive treatment option, particularly for deep and infiltrative lesions not amenable to surgical excision. Early recognition and appropriate imaging, particularly MRI, are essential for establishing the correct diagnosis, guiding treatment, and preventing potential visual complications.

## Patient consent

Written informed consent was obtained from the patient’s legal guardian for publication of this case report and accompanying images, in accordance with institutional ethical standards.
